# Seasonal Diversity of Lactic Acid Bacteria in Artisanal Yoghurt and Their Antibiotic Susceptibility Pattern

**DOI:** 10.1155/2021/6674644

**Published:** 2021-02-12

**Authors:** Lamye Glory Moh, Pamo Tedonkeng Etienne, Kuiate Jules-Roger

**Affiliations:** ^1^Department of Biochemistry, Faculty of Science, University of Dschang, P.O. Box 67, Dschang, Cameroon; ^2^Department of Microbiology, Faculty of Science, University of Yaounde 1, Yaounde, Cameroon; ^3^Department of Animal Production, Faculty of Agronomy and Agricultural Science, University of Dschang, Dschang, Cameroon

## Abstract

The microbiological quality of artisanal yoghurt marketed in some regions of Cameroon was evaluated during the dry and rainy seasons alongside three commercial brands and the susceptibility of isolates to some conventional antibiotics. A total of ninety-six (96) samples were collected, and the microbiological quality was based on the total count of lactic acid bacteria (lactobacilli and cocci) as well as the identification of species present using identification kits. The susceptibility of isolates was determined using the microdilution method. The lactobacillus counts of locally made yoghurts during both seasons were lower than those of the commercial samples. However, there was a general reduction of viable count of lactobacilli within the samples during the rainy season when compared to the dry season while a general increase in the total coccus count was observed during the rainy season except samples from Bamenda which instead decreased. Five (5) *Lactobacillus* species belonging to one genera were identified from 29 lactobacillus isolates. *Lactobacillus bulgaricus* was the highest (64.28%), present in 71.42%, 50.00%, 33.33%, and 33.33% (dry season) compared to 85.71%, 100%, 33.33%, and 25.00% (rainy season) from Bamenda, Dschang, Bafoussam, and commercial, respectively. More so, 14 cocci, 3 coccobacilli, and 1 rod species belonging to 5, 3, and 1 genera were identified, from 74 cocci, 12 coccobacilli, and 3 rod isolates, respectively, with *Streptococcus thermophilus* being the highest (35.55%). However, 93.33% of the lactobacillus isolates were very sensitive to the antibiotics used, while only 20% of cocci were sensitive. This result suggests that the paucity of the appropriate lactic acid bacteria (LAB) and presence of pathogenic LAB caused by the absence of quality control and ignorance might hinder its health benefits and protection offered to consumers with a resultant exposure to high risk of food borne infection and intoxication coupled to the resistant strains.

## 1. Introduction

Yoghurt is a coagulated milk product resulting from the fermentation of lactose in milk by lactic acid bacteria (LAB) (*Lactobacillu*s *delbrueckii* subsp. *bulgaricus* and *Streptococcus salivarius* subsp*. thermophilus*) to liberate lactic acid which is responsible for its unique taste [1, 2]. LAB comprises a heterogeneous group of gram positive bacteria, acid tolerant, generally nonsporulating, nonrespiring rods, or cocci grouped together based on their common metabolic and physiological characteristics [3]. There are about 20 genera within the phylum Firmicutes, and the genera *Aerococcus*, *Carnobacterium*, *Enterococcus*, *Lactobacillus*, *Lactococcus*, *Leuconostoc*, *Oenococcus*, *Pediococcus*, *Streptococcus*, *Tetragenococcus*, *Vagococcus*, and *Weissella* have been considered as the principal LAB [4, 5]. Some of these genera (*Lactococcus* and *Lactobacillus*) are considered safe while members of the genera *Streptococcus* and *Enterococcus* have been known to contain some opportunistic pathogens [6]. These bacteria are widely found in nature, including the gastrointestinal and urogenital tracts of humans [7, 8] and animals [7], environments rich in carbohydrates, decomposing plants, and fermented foods such as cheeses and yoghurts.

Lactic acid, produced by LAB during fermentation, as the major metabolic end product together with other antimicrobial substances, inhibits the growth of unwanted microorganisms [9, 10]. This inhibition increases yoghurt nutritive and sanitary qualities and shelf life, while reducing the risk of food borne diseases [11, 12]. However, the presence of pathogens and spoilage microorganisms in yoghurt without the minimum viable LAB count makes it unsafe to the consumers despite its nutritional composition [13, 14]. Since yoghurt is mostly consumed because of the increasing search for healthy foods, it is very important for it to provide the required health benefits to its consumers [15, 16].

Some LAB present in yoghurt may be resistant to conventional antibiotics, constituting a serious health problem because the pathogenic ones may cause infections in people that may be difficult to be eradicated [17, 18]. According to World Health Organization [17], the rate of emergence of antimicrobial resistance has increased because of improper autoprescription of antibacterial substances. During the recent decades, there has been concern about the possibility of antibiotic resistance spreading in the environment [18]. Therefore, looking for resistant microorganisms in food is essential to ensure food safety, create awareness among common people (producers and consumers), and to protect the health and rights of consumers.

It is generally accepted that yoghurt should contain a minimum of 10^7^ cfu/ml of viable bacteria during consumption [19]. This is not always the case as some have been known to either have bacterial count above or equal [20, 21] or below [22, 23] the recommended dose. More so, when culture or previous day yoghurt used in the fermentation of yoghurt is undefined and used in improper ratio and amount, like the case of artisanal yoghurt, it may contain mixtures of various desirable and undesirable strains of LAB bacteria. As such, the quality of yoghurts may vary with the type of starter culture used [24, 25]. However, having the recommended dose without species identification can be misleading because it is very important to know the exact species present in these yoghurts. In Cameroon, artisanal yoghurts are produced during the rainy and dry seasons and most producers do abandon production during the former season because of cold weather, low consumption, longer fermentation time, and/or difficulties during yoghurt fermentation [14]. This yoghurt is cheaper than the commercial types which in turn are preferred by consumers who have little or no knowledge of its quality [13]. These locally made yoghurts are often prepared without any care of quality control and hygienic conditions due to negligence and ignorance by producers and the competent authorities. More so, they are prepared by these producers using different means and methods without an idea of the advantages and disadvantages of each method. All these, together with seasonal changes, enabled us to conceive this work, which is aimed at comparing the microbiological quality of different yoghurts (artisanal and commercial) produced in some towns of Cameroon in terms of LAB enumeration and species present during the dry and rainy seasons, as well as their susceptibility to some conventional antibiotics.

## 2. Materials and Methods

### 2.1. Collection of Samples

Samples of locally made yoghurts were collected from 15 (dry seasons) and 14 **(**rainy seasons) producers in duplicate at three different occasions from some towns (Bamenda, Bafoussam, and Dschang) of Cameroon. This gave a total of eighty-seven (87) samples, forty-five (45) and forty-two (42) during the dry (November-January) and rainy (May-early August) seasons, respectively. Concurrently, there are three (3) commercial brands of yoghurt: (BB), (AA), and (CC) available in Cameroon were equally collected on the same day from a well-known sale point in Dschang, giving an overall sample size of ninety-six (96). Samples were collected in sterile and labeled containers and transported under aseptic conditions in an ice-packed cooler, at 4-7°C to the Research Unit of Microbiology and Antimicrobial Substances (RUMAS), Faculty of Science, University of Dschang.

### 2.2. Microbiological Analysis

#### 2.2.1. Preparation of Materials

All media were obtained in dehydrated forms and prepared according to the manufacturer's instructions. Glass wares such as Petri dishes, test tubes, pipettes, flasks, and bottles were sterilized in a hot oven at 170°C for two hours [26].

#### 2.2.2. Preparation of Serial Dilutions

This was done according to A.P.H.A, [27] during which one milliliter (1 ml) of yoghurt from a homogenous sample was ten folds serially diluted into 9 ml of sterile distilled water to obtain a series of eight dilutions ranging from 10^−1^ to 10^−8^. Fifty microliters (50 *μ*l) of diluted samples were spread over prepared dried plates with different media.

#### 2.2.3. Enumeration of Lactic Streptococci and Other Cocci

M17 agar (Qingdao-China) was used to enumerate *Streptococcus* sp. and other cocci in yoghurt samples and incubated aerobically at 37°C for 48 hours according to Torriani et al. [28].

#### 2.2.4. Enumeration of Lactobacilli

MRS Rogosa agar (Micromaster Laboratories Pvt. Ltd, Maharashtra-India) was used for enumeration of lactobacilli according to Tharmaraji and Shah [29]. Plates were incubated under microaerophilic condition at 37°C for 48 hours.

Colonies from the cultured plates were differentiated and purified using the streak method. Purified colonies were prepared in their respective broth, MRS broth (Micromaster Laboratories Pvt. Ltd, Maharashtra-India) for lactobacilli and M-17 broth (Qingdao-China) for cocci.

### 2.3. Identification of Lactic Acid Bacteria

Gram positive and catalase negative bacteria were examined microscopically (cell morphology and arrangements). The rods and coccus bacteria were presumptively identified as LAB according to Gerhartdt et al. [30]. The ability to ferment carbohydrate substrates was studied using the API 50 CH galleries and API 50 CHL medium(lactobacilli), and API 20 Strep (cocci) (BioMerieux, Marcy l'Etoile, France) system, which enabled the identification of LAB isolates to species level. All tests were performed according to the manufacturer's instructions. The API LAB PLUS database (BioMerieux, SA) and accompanying computer software were used to interpret the result obtained.

### 2.4. The Susceptibility of Isolates (LAB) to Conventional Antibiotics

#### 2.4.1. Chemicals for Susceptibility Testing

Tetracycline (TET), ciprofloxacin (CIP), chloramphenicol (CHL), ampicillin (AMP), streptomycin (STR), gentamycin (GEN), erythromycin (ERY), and norfloxacin (NOR) (Sigma-Aldrich, St Quentin Fallavier, France) were used as reference antibiotics (RA). *ρ*-Iodonitrotetrazolium chloride (INT, Sigma-Aldrich) was used as a microbial growth indicator [31, 32]. The antibiotics used in this study were selected on the basis of their mechanisms of action, targeting different sites or processes although duplicates were included in some cases.

#### 2.4.2. Microbial Strains and Culture Media

All microorganisms were obtained from the yoghurt samples used in this study. MRS agar (for lactobacilli) and M-17 agar (lactic streptococci and other cocci) were used to activate the tested LAB isolates. The minimum inhibitory concentration (MIC) and minimum microbicidal concentration (MBC) determinations on the tested bacteria were conducted using rapid INT colorimetric assay.

#### 2.4.3. Determination of MIC and MBC by INT Colorimetric Assay

The MICs were obtained by using the rapid INT colorimetric assay according to described methods [31] with some modifications [33]. The antibiotics were dissolved in dimethylsufoxide (DMSO) solution, introduced into the first wells of the microtitre plates and serially diluted two folds with Mueller Hinton Broth (MHB) to a final volume of 100 *μ*l. One hundred microliters (100 *μ*l) of inoculum 1.5 × 10^6^ cfu/ml prepared in the broth was added [33]. The plates were covered with a sterile plate sealer and incubated at 37°C for 18 h. The MICs of samples were detected following addition of INT (40 *μ*l of 0.2 mg/ml) and incubation at 37°C for 30 min. Viable bacteria reduced the yellow dye to pink. MIC was defined as the lowest sample concentration that prevented the color change of the medium and exhibited complete inhibition of microbial growth [31]. MBCs were determined by adding 50 *μ*l aliquots of the preparations, which did not show any growth after incubation and which did not receive INT, to 150 *μ*l of broth. These preparations were incubated at 37°C for 48 h. The MBC was regarded as the lowest concentration of antibiotic, which did not produce a color change after addition of INT as mentioned above [33]. The final concentration of DMSO was lower than 2.5% and has been known not to affect the microbial growth [34]. The assays were performed in triplicate and repeated thrice.

### 2.5. Statistical Analysis

Analyzable data were subjected to the one-way analysis of variance (ANOVA), and differences between samples at *p* ≤ 0.05 were determined by Waller-Duncan test using the Statistical Package for the Social Sciences (SPSS) version 11.0. The results were expressed (where appropriate) as mean ± standard deviation of the replicates.

## 3. Results and Discussion

### 3.1. Lactic Acid Bacteria Count

#### 3.1.1. Lactobacillus Count

Samples PA, MR, and MB (Bamenda), P (Dschang), K (Bafoussam), and BB (commercial 2) were completely void of lactobacilli during the dry and rainy season (except MB) as shown in Table 1. Just as during the dry season, lactobacillus counts of all artisanal yoghurt samples were not significantly different (*p* > 0.05) from those of the commercial samples, and within the samples, there was a general reduction of viable count during the rainy season when compared to the dry season (Table 1). Within the samples (commercial and artisanal), the reduction in colony counts was generally 10 folds, and most of the yoghurt samples had very low counts of lactobacilli. The low count observed during the rainy season could probably be due to the fact that *Lactobacillus bulgaricus* needs a particular temperature for proper growth. Temperatures below or above will lead to failure, and as such, temperature fluctuations during this season might negatively affect the survival of these microorganisms.

#### 3.1.2. Coccus Count

The total coccus count of 66.66%, 50%, 50%, and 0% of samples from Dschang, Bamenda, Bafoussam, and commercial, respectively, did not vary significantly (*p* > 0.05) among themselves during the dry season (Table 1). Generally, there was no significant difference (*p* > 0.05) between the locally made or artisanal yoghurts and the commercial yoghurt samples except for samples from Bamenda which were significantly lower (*p* < 0.05). More so, samples from Dschang and Bafoussam were significantly higher (*p* < 0.05) than the commercials during the rainy season (Table 1). Fifty percent (50%) and thirty percent (30%) of the artisanal yoghurts had coccus counts significantly lower than those of the commercial samples during the rainy and dry seasons, respectively. There was a general increase in the total coccus count during the rainy season when compared to those obtain during the dry season (Table 1) with the exception of samples from Bamenda which did not vary significantly (*p* > 0.05). Summarily, samples from Bamenda had the lowest coccus counts during both seasons. During the rainy season, the turnover of yoghurt is lower because of ignorance; a good number of people consume it just to quench taste; as such, there is little or limited consumption during this season. This could lead to a longer selling/storage time, and hence, temperature fluctuations caused by daily movement to and from the market, thus a favorable environment for the cocci to multiply.

Yoghurt should contain 10^7^ viable cells of LAB (lactobacilli and cocci) per milliliter in the finished product in order to produce the desired health benefits [1, 35]. This standard was met in 26.67% and 35.71% of all the samples collected during the dry and rainy seasons, respectively, while it was 0% and 33.33% for commercial samples collected during the two seasons, respectively. Variations in total viable bacterial counts (lactobacilli and cocci) among artisanal yoghurt samples might be due to the use of left-over artisanal and/or commercial yoghurt for fermentation in improper ratio and amount which contains heterogeneous mixture of LAB coupled to the absence of quality control. Variations may also be caused by temperature fluctuations as a result of unstable electricity supply. This variation was also observed by Masud et al. [24].

### 3.2. Lactic Acid Bacteria Species

#### 3.2.1. Lactobacillus Species

Twenty-nine (29) lactobacillus isolates obtained from ninety-six (96) yoghurt samples were identified to species level with 13 and 15 isolates during the dry (D.S) and rainy (R.S) seasons, respectively (Table 2). These isolates were further distributed as follows: 12 (5 D.S and 7 R.S), 3 (2 D.S and 1 R.S), 6 (3 D.S and 3 R.S), and 7 (3 D.S and 4 R.S) from Bamenda, Dschang, Bafoussam, and commercial, respectively. Five (5) *Lactobacillus* species belonging to one genera were identified, with the number of occurrences indicated in parentheses: *Lactobacillus bulgaricus* (18), *Lactobacillus viridescens* (2), *Lactobacillus fermentum* (4), *Lactobacillus lactis* (2), and *Lactobacillus cellobiosus* (3) (Table 3).

It was noticed that although these samples had lactobacilli as shown by colony count on MRS agar, it was revealed after identification that 30.77% of samples had lactobacilli entirely different from *L. bulgaricus*. The other samples had either *L. bulgaricus* or mixed with other lactobacilli (Table 2). Among the samples that had lactobacilli, 83.33%, 50%, 33.33%, and 50% from Bamenda, Dschang, Bafoussam, and commercials had *L. bulgaricus*, respectively (Table 2). Out of 17 yoghurt samples, 13 had *Lactobacillus* sp. with *L. bulgaricus* found in 9 and *L. viridescens* and *L. fermentum* in 2; meanwhile, *L. lactis* and *L. cellobiosus* were found in just one each. From the 29 isolates, *L. bulgaricus* was the highest (64.28%), present in 71.42%, 50.00%, 33.33%, and 33.33% (dry season) compared to 85.71%, 100%, 33.33%, and 25.00% (rainy season) from Bamenda, Dschang, Bafoussam, and commercial, respectively. This was followed by *L. fermentum* (14.28%), absent in samples from Bamenda, Dschang, and commercial but representing 66.67% of isolates from Bafoussam during both seasons. The rest had the following percentages: *L. cellobiosus* (10.71%), *L. lactis*, and *L. viridescens* (7.14%) as shown in Table 2.

*L. fermentum*, *L. lactis*, and *L. cellobiosus* were mostly found in the commercial yoghurts except *L. fermentum* which was present in some yoghurt samples from Bafoussam. Although some may be useful, their presence in yoghurt could be accidental. They surely entered into these yoghurts as contaminants. *L. fermentum* is a heterofermentative LAB, when allowed to grow in significant numbers in yoghurt can cause defects such as bloated packaging because of the production of other acids and CO_2_. *L. cellobiosus* is a biotype of *L. fermentum* since they are closely related [36]. Meanwhile, *L. viridescens* is an ubiquitous organism found in meat, plants, and meat products [37, 38] associated with discolorations [39] and might have entered into yoghurt samples through unhygienic practices during production. It is also considered a potent spoilage organism, as shelf life is reduced due to deterioration caused by this pathogen.

#### 3.2.2. Cocci, Coccobacilli, and Rod Species

Seventy-four (74) cocci, 12 coccobacilli, and 3 rods from ninety-six (96) yoghurt samples were identified to species level (Table 4). The isolates were distributed as follows: cocci (33 (16 D.S and 17 R.S)), 11 (6 D.S and 5 R.S), 17 (9 D.S and 8 R.S), and 13 (6 D.S and 7 R.S); coccobacilli (11 (6 D.S and 5 R.S)), 1 (1 D.S and 0 R.S), 0, and 0; rods (2 (1 D.S and 1 R.S)), 1 (0 D.S and 1 R.S), 0, and 0 from Bamenda, Dschang, Bafoussam, and commercial, respectively. Fourteen (14) cocci, 3 coccobacilli, and 1 rod species belonging to 5, 3, and 1 genera were identified, respectively, with the number of occurrences indicated in parentheses: *Streptococcus thermophilus* (32), *Streptococcus constellatus* (2), *Streptococcus canis* (1), *Streptococcus acidomonimus* (4), *Streptococcus uberis* (1), *Streptococcus bovis* 1 (1), *Enterococcus faecium* (3), *Enterococcus durans* (6), *Enterococcus avium* (3), *Aerococcus viridans* 1 (10), *Aerococcus viridans* 2 (8), *Aerococcus viridans* 3 (1), *Lactococcus lactis* subsp. *lactis* (2), *Leuconostoc* sp. (coccobacilli) (10), *Listeria* sp. (rod) (3), *Abiotrophia defectiva* (coccobacilli) (1), *Gardnerella vaginalis* (coccobacilli) (1), and *Gemella haemolysans* (1) (Table 5).

Besides *S. thermophilus*, so many other cocci and even coccobacilli and rods of which some can cause diseases in humans were isolated, indicating a high level of bad production practices. The season or time of the year had no effect on *S. thermophilus*, which was the highest represented isolate (35.55%), present in 76.47% of samples. All samples from Bamenda and Dschang had *S. thermophilus*, but only 50% and 33.33% of samples from Bafoussam and commercial brands, respectively, had it. This was followed by *A. viridans* (21.11%), present in 22.22% of samples during the dry and rainy seasons. *A. viridans* occupied 31.81%, 25%, 11.11%, and 16.67% (dry season) as well as 29.16%, 0.00%, 25%, and 14.28% (rainy season) from Bamenda, Dschang, Bafoussam, and commercial, respectively (Table 4). Apart from that, enterococci (*Enterococcus faecium*, *Enterococcus durans*, and *Enterococcus avium*) was the third abundant isolate (13.33%) with 0.00%, 12.5%, 22.22%, and 50% (dry season) as well as 4.17%, 16.67%, 12.50%, and 42.86% (rainy season) occurrences in samples from Bamenda, Dschang, Bafoussam, and commercial, respectively (Table 4). It had a lower percentage of occurrences in samples from Bamenda during both seasons (Table 5). *Leuconostoc* sp. (coccobacilli) was the fourth with 11.11% of occurrence and absent in samples from Bafoussam and commercial brands (4). It represented 18.18% and 20.83% of isolates from Bamenda during the dry and rainy seasons, respectively, but only present during the dry season in some samples from Dschang (25%). Lastly, *Lactococcus lactis* subsp. *lactis* was found in just two samples Ce (Bafoussam) and CC (commercial yoghurt) representing 2.22% of isolates (Table 4).

Apart from *S. thermophilus* which is used as a starter in the production of yoghurt as well as the production of fermented dairy products [40], the rest of the *Streptococcus* species (*Streptococcus constellatus*, *Streptococcus canis*, *Streptococcus acidomonimus*, *Streptococcus uberis*, and *Streptococcus bovis*), representing 8.88% of isolates, have been associated with infections in humans and even animals [41]. However, the abundance of *A*. *viridans* during the dry season may be attributed to the dusty environment during this period of the year since it is a saprophytic microorganism commonly found in the air of occupied places and in dust [42]. *Enterococcus* sp., found almost every in the environment when present in yoghurt, indicates neglect in sanitary control measures during production and is also implicated in food poisoning outbreaks [43–45]. They can even survive the unfavorable low pH environment of yoghurt [46]. Some strains have probiotic traits [47, 48] like *E. faecium* [49], whereas others (*E. faecium*, *E. durans*, and *E. avium*) are the causative agents for serious infections in humans [50, 51]. *Leuconostoc* sp. (coccobacilli), a heterofermentative LAB, is involved in a large number of spontaneous fermentations of food products and contributes to the nonstarter lactic acid bacteria (NSLAB) populations of dairy environments [52] with antimicrobial action against microorganisms [53]. More so, *Lc*. *lactis* subsp. *lactis* present in just two samples Ce (artisanal from Bafoussam) and CC (commercial yoghurt) has also been used on a large scale in food industries for the manufacture of various fermented dairy products such as sour milk [54]. Since the two samples (Ce and CC) were void of *L*. *bulgaricus and S*. *thermophilus*, it might be concluded that these producers (CC and Ce) intentionally used it to acidify their fermented products. The rest of the cocci (*Gemella haemolysans*), rods (*Listeria* sp.), and coccobacilli (*Abiotrophia defectiva* and *Gardnerella vaginalis*) which were also isolated from some of the artisanal yoghurt samples have been known to be pathogenic to man and even animals [55, 56].

### 3.3. Susceptibility of Lactic Acid Bacteria Isolates to Conventional Drugs

#### 3.3.1. Susceptibility of Cocci, Coccobacilli, and Rod Isolates to Some Conventional Antibiotics

The minimal inhibitory concentration (MIC) is the lowest antibiotic concentration that inhibits visible bacterial growth after overnight incubation [57]. It is considered that when MICs are ≥8 *μ*g/ml, the bacteria may be considered as “moderately resistant”; when MICs are above 32 *μ*g/ml, it may be classified as “clinically resistant” to the antibiotic [58]. Based on this scale, 53.33% (8/15) of the coccus isolates were clinically resistant to all the tested antibiotics and 26.67% (4/15) were resistant to at least 4 antibiotics, while only 20% (3/15) were sensitive to most of the antibiotics. More so, 100% of coccobacilli (3/3) and rods (1/1) were clinically resistant to all the antibiotics used (Table 6).

The profiles of antimicrobial susceptibility of LAB have been documented in many countries [59, 60]. Most of the *Streptococcus* sp. were sensitive to a majority of the antibiotics. This was in line with the works of other researchers who stated that most members of the *Streptococcus* sp. are susceptible to the beta-lactams, macrolides, and chloramphenicol [61, 62]. Only the MBC values of 36.84% of isolates were determined (Table 6). The ratio MBC/MIC was generally below 4, indicating that these conventional antibiotics exerted bactericidal effects [63] except *Streptococcus constellatus* whose MBC/MIC ratio was above 4 in most cases hence bacteriostatic. Most *Aerococcus* sp. were resistant and this corroborated other results that they are resistant to chloramphenicol, quinolones, ampicillin, and gentamicin [64]. It has also been reported that *Leuconostoc* sp. (coccobacilli) are usually resistant to gentamycin and streptomycin while most species are sensitive to chloramphenicol, erythromycin, and tetracycline [60]. In this study, the species was resistant to all the antibiotics used. The enterococcus species (13.33%) being the third abundant coccus isolate obtained in this study are controversial species that should not be used for probiotic applications, because of its notable resistance to some of the widely used antibiotics. Moreover, they are known to be resistant to most antibiotics including aminoglycosides, macrolides, chloramphenicol, tetracyclines, and quinolones [65]. Recent studies have focused on enterococci due to their increasing antibiotic resistance [66] although some members of this genus have been known to possess health-promoting effects and technological properties [67]. In addition, this genus can transfer the antibiotic resistant-encoding gene to pathogens, for example, between enterococci and staphylococci [68], spreading antibiotic resistance into the environment and food chains [65, 69], which is a serious public health problem. The rest of the cocci (*Gemella haemolysans*), rods (*Listeria* sp.), and coccobacilli (*Abiotrophia defectiva* and *Gardnerella vaginalis*) which have been known to be pathogenic to man and even animals were highly resistant to all antibiotics used. This can be very dangerous since they may cause infections in humans that are difficult to be treated.

#### 3.3.2. Susceptibility of Lactobacillus Isolates to Some Conventional Antibiotics

Unlike the cocci, the lactobacilli were generally very sensitive to the conventional antibiotics except *L. fermentum* (C3 II), which was resistant to all the antibiotics at the tested concentration (Table 7). However, 91.67% (14/15) of isolates were resistant to norfloxacin and streptomycin but sensitive to ciprofloxacin, erythromycin, tetracycline, and ampicillin while only 20.00% (3/15) were resistant to gentamycin and chloramphenicol. Generally, 93.33% (14/15) of the isolates were very sensitive while only 6.67% (*L. fermentum*) were resistant (Table 7). The MBC values were also obtained, and the MBC/MIC ratio was generally below 4 as in the case of cocci, meaning that the conventional antibiotics exerted a bactericidal effect on the lactobacilli. Furthermore, the high sensitivity of lactobacilli to antibiotics is in line with results of other works, which stated that they are generally sensitive to inhibitors of protein synthesis such as chloramphenicol, erythromycin, and tetracycline as well as *β*-lactamase inhibitors (ampicillin) [70, 71]. However, lactobacilli have been known to be intrinsically resistant to aminoglycosides and fluoroquinolones [72]. This explains the 100% resistance observed with norfloxacin and streptomycin.

## 4. Conclusion

These results suggest that yoghurt production is best during the dry season because of appropriate environmental temperatures and high consumption. The paucity of the appropriate LAB and presence of pathogenic LAB caused by the absence of quality control and ignorance might hinder the health benefits and protection, which the food should provide to the consumers, thereby exposing them to high risk of food borne infection and intoxication coupled to the resistant isolates. The relatively high level of resistance (especially the cocci) to antimicrobial agents constitutes a major threat to public health as it may spread bacterial resistance among the populace who come in contact with these yoghurts. Moreover, the sensitivity of lactobacilli to these antibiotics suggests that yoghurt should be avoided during antibiotic treatment.

## Figures and Tables

**Table 1:** Lactic acid bacteria counts of yoghurt during the dry and rainy seasons as a function of place of production and sample.

Location	Samples	Lactic acid bacteria colony counts (log10 cfu/ml)			
		Dry season		Rainy season	
		Lactobacillus counts	Coccus counts	Lactobacillus counts	Coccus counts
Dschang	V	3.46 ± 0.24^c^	5.24 ± 0.18^d^	/	/
Dschang	P	0.00 ± 0.00^a^	7.30 ± 0.05^h^	0.00 ± 0.00^a^	9.70 ± 0.04^l^
Dschang	G	4.75 ± 0.05^g^	5.31 ± 0.02^d^	3.98 ± 0.07^ef^	6.26 ± 0.04^h^
	Mean	2.74 ± 2.13*^α^*	5.95 ± 1.01*^β^*	1.99 ± 2.18*^α^*	7.98 ± 1.88*^γ^*
Bamenda	NR	4.09 ± 0.01^f^	4.70 ± 0.19^b^	3.02 ± 0.21^cd^	3.48 ± 0.12^b^
Bamenda	S	2.94 ± 0.18^b^	4.36 ± 0.12^a^	4.15 ± 0.13^ef^	3.83 ± 0.04c
Bamenda	D	3.85 ± 0.05^e^	4.78 ± 0.02^bc^	4.18 ± 0.07^ef^	4.06 ± 0.03^d^
Bamenda	MR	0.00 ± 0.00^a^	5.58 ± 0.01^e^	0.00 ± 0.00^a^	3.11 ± 0.28^a^
Bamenda	MB	0.00 ± 0.00^a^	4.30 ± 0.34^a^	3.61 ± 0.07^de^	4.01 ± 0.02^d^
Bamenda	PA	0.00 ± 0.00^a^	7.33 ± 0.09^h^	0.00 ± 0.00^a^	5.86 ± 0.03^g^
Bamenda	SY	4.74 ± 0.03^g^	4.25 ± 0.03^a^	4.08 ± 0.09^ef^	4.24 ± 0.04^e^
Bamenda	PV	3.89 ± 0.01^e^	4.30 ± 0.34^a^	3.18 ± 0.06^cd^	4.01 ± 0.02^d^
	Mean	2.44 ± 1.98*^α^*	4.95 ± 1.02*^α^*	2.78 ± 1.69*^α^*	4.07 ± 0.77*^α^*
Bafoussam	T	4.07 ± 0.15^f^	5.32 ± 0.04^d^	3.76 ± 0.12^de^	8.67 ± 0.06^k^
Bafoussam	Ce	3.67 ± 0.09^d^	5.24 ± 0.18^d^	2.60 ± 0.30^c^	9.71 ± 0.04^lm^
Bafoussam	C	5.14 ± 0.04^h^	7.72 ± 0.02^i^	1.79 ± 1.55^b^	9.84 ± 0.03^m^
Bafoussam	K	0.00 ± 0.00^a^	7.76 ± 0.02^i^	0.00 ± 0.00^a^	9.68 ± 0.05^l^
	Mean	3.22 ± 2.02*^α^*	6.51 ± 1.28*^β^*	2.04 ± 1.58*^α^*	9.47 ± 0.49*^δ^*
Commercial 1	AA	5.19 ± 0.08^hi^	6.20 ± 0.01^f^	4.58 ± 0.08^f^	5.13 ± 0.19^f^
Commercial 2	BB	0.00 ± 0.00^a^	6.74 ± 0.03^g^	0.00 ± 0.00^a^	7.78 ± 0.01^j^
Commercial 3	CC	5.31 ± 0.10^i^	4.94 ± 0.04^c^	4.25 ± 0.15^ef^	6.82 ± 0.03^i^
	Mean	3.50 ± 2.62*^α^*	5.96 ± 0.79*^β^*	2.94 ± 2.21*^α^*	6.58 ± 1.16*^β^*

Values are mean ± SD of 3 determinants. ^a,b,c,d,e,f,g,h,i,j,k,l,m^Comparison of different samples for each microbial parameter. Along the column, values with the same letter are not significantly different (*p* > 0.05). ^*α*,*β*,*γ*,*δ*^Comparison of mean of all samples from different places and seasons, mean with the same Greek alphabet are not significantly different (*p* > 0.05); /: absence of producer.

**Table 2:** Lactobacillus species found in yoghurt samples during the dry and rainy season.

Place	Name of isolate	Producers	
		Dry season	Rainy season
Bamenda	*Lactobacillus bulgaricus*	NR, D, PV, S, SY	NR, D, PV, S, MB, SY
	*Lactobacillus viridescens*	/	D
Dschang	*Lactobacillus bulgaricus*	G	G
	*Lactobacillus viridescens*	V	/
Bafoussam	*Lactobacillus bulgaricus*	T	T
	*Lactobacillus fermentum*	Ce, C	Ce, C
Commercials	*Lactobacillus bulgaricus*	AA	AA
	*Lactobacillus lactis*	AA	AA
	*Lactobacillus cellobiosus*	CC	CC

/: absence of lactobacilli.

**Table 3:** The distribution of lactobacillus species as a function of place.

	Yoghurt samples/season								Frequency of occurrence	% of occurrence	Frequency of occurrence	% of occurrence
Lactobacilli isolated from yoghurt	Bamenda		Dschang		Bafoussam		Commercial					
	Dry season	Rainy season	Dry season	Rainy season	Dry season	Rainy season	Dry season	Rainy season	Dry season	Dry season	Rainy season	Rainy season
*Lactobacillus bulgaricus*	**+**	**+**	**+**	**+**	**+**	-	**+**	**+**	4	100	3	75
*Lactobacillus viridescens*	**+**	-	-	**+**	-	-	-	-	1	25	1	25
*Lactobacillus fermentum*	-	-	-	-	-	**+**	-	-	0	00	1	25
*Lactobacillus lactis*	-	-	-	-	-	-	**+**	**+**	1	25	1	25
*Lactobacillus cellobiosus*	-	-	-	-	-	-	**+**	**+**	1	25	1	25

+: positive; -: negative.

**Table 4:** Cocci, coccobacilli, and rod species found in yoghurt samples during the rainy and dry season.

Place	Name of isolate	Producers	
		Dry season	Rainy season
Bamenda	*Streptococcus thermophilus*	NR, MR, PV, SY, D, S, MB	NR, MR, PV, SY, D, S, MB
	*Aerococcus viridans 2*	NR, SY	NR, PV, SY
	*Aerococcus viridans 1*	D, PV, S, MB	/
	*Enterococcus avium*	/	PV
	*Listeria* sp. (rods)	NR	S
	*Leuconostoc* sp. (coccobacilli)	MR, PV, SY	NR, MR, D, PV, MB
	*Streptococcus bovis 1*	PV	/
	*Gemella haemolysans*	S	/
	*Gardnerella vaginalis* (coccobacilli)	/	NR
	*Abiotrophia defective* (coccobacilli)	S	/
Dschang	*Streptococcus thermophilus*	P, V, G	P, G
	*Streptococcus acidomonimus*	/	P
	*Aerococcus viridans 3*	P	/
	*Aerococcus viridans 1*	V	/
	*Enterococcus durans*	P	G
	*Leuconostoc* sp. (coccobacilli)	V, G	/
	*Listeria* sp. (rods)	/	P
Bafoussam	*Streptococcus thermophilus*	C, K	C, K
	*Streptococcus acidomonimus*	T, Ce	T
	*Streptococcus uberis*	/	C
	*Enterococcus durans*	/	C
	*Lactococcus lactis* subsp. *lactis*	/	Ce
	*Streptococcus canis*	K	/
	*Aerococcus viridans 1*	T	C
	*Aerococcus viridans 2*	/	Ce
	*Enterococcus durans*	C	/
	*Enterococcus faecium*	C	/
Commercial	*Streptococcus thermophilus*	AA	AA
	*Streptococcus constellatus*	BB	BB
	*Lactococcus lactis* subsp. *lactis*	/	CC
	*Enterococcus durans*	AA	AA
	*Enterococcus faecium*	AA	AA
	*Enterococcus avium*	CC	CC

/: absence of lactic streptococci and other cocci.

**Table 5:** The distribution of cocci, coccobacilli, and rod species as a function of locality and season.

	Yoghurt samples/season								Frequency of occurrence	% of occurrence	Frequency of occurrence	% of occurrence
Cocci, coccobacilli, and rod species isolated from yoghurt	Bamenda		Dschang		Bafoussam		Commercial					
	Dry season	Rainy season	Dry season	Rainy season	Dry season	Rainy season	Dry season	Rainy season	Dry season	Dry season	Rainy season	Rainy season
*Streptococcus thermophilus*	**+**	**+**	**+**	**+**	**+**	**+**	**+**	**+**	4	100	4	100
*Streptococcus constellatus*	-	-	-	-	-	-	**+**	**+**	1	25	1	25
*Streptococcus canis*	-	-	-	-	**+**	-	-	-	1	25	0	00
*Streptococcus acidomonimus*	-	-	-	**+**	**+**	**+**	-	-	1	25	2	50
*Streptococcus uberis*	-	-	-	-	-	**+**	-	-	0	00	1	25
*Streptococcus bovis 1*	**+**	-	-	-	-	-	-	-	1	25	0	00
*Enterococcus faecium*	-	-	-	-	**+**	-	**+**	**+**	2	50	1	25
*Enterococcus durans*	-	-	**+**	**+**	**+**	**+**	**+**	**+**	3	75	3	75
*Enterococcus avium*	-	**+**	-	-	-	-	-	-	0	00	1	25
*Aerococcus viridans 1*	**+**	**+**	**+**	-	**+**	**+**	-	-	3	75	2	50
*Aerococcus viridans 2*	**+**	**+**	-	-	-	**+**	**+**	**+**	2	50	3	75
*Aerococcus viridans 3*	-	-	**+**	-	-	-	-	-	1	25	0	00
*Lactococcus lactis* subsp. *lactis*	-	-	-	-	-	**+**	-	**+**	0	00	2	50
*Leuconostoc* sp. (coccobacilli)	**+**	**+**	**+**	-	-	-	-	-	2	50	1	25
*Listeria* sp. (rods)	**+**	**+**	-	**+**	-	-	-	-	1	25	2	50
*Abiotrophia defective* (coccobacilli)	**+**	-	-	-	-	-	-	-	1	25	0	00
*Gardnerella vaginalis* (coccobacilli)	**+**	**+**	-	-	-	-	-	-	1	25	1	25
*Gemella haemolysans*	**+**	-	-	-	-	-	-	-	1	25	0	00

+: positive; -: negative.

**Table 6:** The susceptibility of cocci, coccobacilli, and rod species to some conventional antibiotics.

Coccus species	Parameter	Antibiotic							
		GEN	CIP	ERY	TET	NOR	AMP	STRE	CHL
*Gemella haemolysans*	MIC	>256	>128	>256	>256	>256	>256	>256	>128
	MBC	ND	ND	ND	ND	ND	ND	ND	ND
*Gardnerella vaginalis* (coccobacilli)	MIC	>256	>128	>256	>256	>256	>256	>256	>128
	MBC	ND	ND	ND	ND	ND	ND	ND	ND
*Streptococcus constellatus*	MIC	16	<1	>256	<2	16	>256	>256	>128
	MBC	64	2	ND	<2	64	ND	ND	ND
*Enterococcus faecium*	MIC	>256	>128	>256	>256	>256	>256	>256	>128
	MBC	ND	ND	ND	ND	ND	ND	ND	ND
*Enterococcus avium*	MIC	>256	>128	>256	>256	>256	>256	>256	>128
	MBC	ND	ND	ND	ND	ND	ND	ND	ND
*Streptococcus canis*	MIC	32	<1	>256	>256	8	<2	256	4
	MBC	256	2	ND	ND	128	<2	/	8
*Enterococcus durans*	MIC	>256	>128	>256	>256	>256	>256	>256	>128
	MBC	ND	ND	ND	ND	ND	ND	ND	ND
*Streptococcus acidomonimus*	MIC	<2	<1	128	<2	<2	>256	4	32
	MBC	128	128	/	64	<2	ND	128	/
*Lactococcus lactis* subsp. *lactis*	MIC	<2	<1	<2	<2	<2	<2	32	4
	MBC	8	<1	<2	<2	<2	16	/	32
*Aerococcus viridans 1*	MIC	256	>128	>256	>256	>256	>256	>256	32
	MBC	/	ND	ND	ND	ND	ND	ND	/
*Aerococcus viridans 2*	MIC	<2	<1	<2	<2	32	8	64	32
	MBC	8	8	64	<2	128	32	256	64
*Aerococcus viridans 3*	MIC	>256	>128	>256	>256	>256	>256	>256	>128
	MBC	ND	ND	ND	ND	ND	ND	ND	ND
*Streptococcus uberis*	MIC	>256	>128	>256	>256	>256	>256	>256	>128
	MBC	ND	ND	ND	ND	ND	ND	ND	ND
*Abiotrophia defectiva* (coccobacilli)	MIC	>256	>128	>256	>256	>256	>256	>256	>128
	MBC	ND	ND	ND	ND	ND	ND	ND	ND
*Streptococcus bovis 1*	MIC	>256	>128	>256	>256	>256	>256	>256	>128
	MBC	ND	ND	ND	ND	ND	ND	ND	ND
*Leuconostoc* sp. (coccobacilli)	MIC	>256	>128	>256	>256	>256	>256	>256	>128
	MBC	ND	ND	ND	ND	ND	ND	ND	ND
*Listeria* sp. (rod)	MIC	>256	>128	>256	>256	>256	>256	>256	>128
	MBC	ND	ND	ND	ND	ND	ND	ND	ND
*Streptococcus thermophilus* (*MB4 I*)	MIC	<2	<1	128	8	8	32	16	4
	MBC	<2	4	256	>256	16	64	64	64
*Streptococcus thermophilus* (*C2 I*)	MIC	8	<1	<2	<2	16	<2	32	4
	MBC	8	<1	16	32	32	<2	/	16

GEN: gentamycin; CIP: ciprofloxacin; ERY: erythromycin; TET: tetracycline; NOR: norfloxacin; AMP: ampicillin; STRE: streptomycin; CHL: chloramphenicol; /: no data; ND: not determined.

**Table 7:** The susceptibility of lactobacillus species to some conventional antibiotics.

Lactobacillus species	Parameter	Antibiotic							
		GEN	CIP	ERY	TET	NOR	AMP	STRE	CHL
*Lactobacillus bulgaricus* (*SY5 I*)	MIC	<2	<1	<2	<2	<2	<2	16	2
	MBC	<2	<1	<2	<2	64	<2	64	2
*Lactobacillus viridescens* (*D5 II*)	MIC	4	2	4	<2	16	<2	16	2
	MBC	4	2	8	<2	16	<2	32	8
*Lactobacillus bulgaricus* (*NR4 II*)	MIC	8	<1	<2	<2	16	<2	64	8
	MBC	16	2	<2	<2	32	<2	128	16
*Lactobacillus viridescens* (*V4 II*)	MIC	<2	<1	<2	<2	16	<2	32	<1
	MBC	<2	<1	64	<2	32	64	64	64
*Lactobacillus fermentum* (*Ce1 II*)	MIC	4	<1	<2	<2	8	<2	8	<1
	MBC	4	2	<2	<2	16	<2	32	4
*Lactobacillus fermentum* (*C3 II*)	MIC	>256	>128	>256	>256	>256	>256	>256	>128
	MBC	ND	ND	ND	ND	ND	ND	ND	ND
*Lactobacillus cellobiosus* (*CC6 III*)	MIC	4	2	<2	<2	16	<2	32	4
	MBC	4	2	<2	<2	16	4	32	4
*Lactobacillus cellobiosus* (*CC7 III*)	MIC	<2	<1	<2	<2	16	<2	<2	<1
	MBC	4	<1	<2	4	16	<2	8	8
*Lactobacillus lactis* (*AA5 II*)	MIC	4	<1	<2	<2	32	<2	32	2
	MBC	8	2	<2	<2	32	<2	32	2
*Lactobacillus bulgaricus* (*AA1 II*)	MIC	4	<1	<2	<2	16	<2	16	2
	MBC	4	<1	<2	<2	16	<2	32	8
*Lactobacillus bulgaricus* (*MB5 II*)	MIC	<2	<1	<2	<2	16	<2	32	4
	MBC	4	2	<2	<2	32	<2	64	8
*Lactobacillus bulgaricus* (*PV8 I*)	MIC	4	<1	<2	<2	16	<2	16	4
	MBC	4	8	8	<2	32	<2	32	16
*Lactobacillus bulgaricus* (*T2 I*)	MIC	4	2	<2	<2	16	<2	32	2
	MBC	8	8	<2	8	32	<2	64	8
*Lactobacillus bulgaricus* (*D1 II*)	MIC	8	<1	<2	<2	16	<2	32	8
	MBC	8	2	<2	<2	32	<2	128	128
*Lactobacillus bulgaricus* (*S2 I*)	MIC	4	<1	<2	<2	16	<2	32	4
	MBC	8	4	4	<2	32	<2	64	16

GEN: gentamycin; CIP: ciprofloxacin; ERY: erythromycin; TET: tetracycline; NOR: norfloxacin; AMP: ampicillin; STRE: streptomycin; CHL: chloramphenicol; ND: not determined.

## Data Availability

The data used to support the findings of this study are included within the article.
